# Predictors of lost to follow-up in a “test and treat” programme among adult women with high-risk sexual behavior in Kampala, Uganda

**DOI:** 10.1186/s12889-020-8439-9

**Published:** 2020-03-18

**Authors:** Onesmus Kamacooko, Yunia Mayanja, Daniel Bagiire, Gertrude Namale, Christian Holm Hansen, Janet Seeley

**Affiliations:** 1 MRC/UVRI & LSHTM Uganda Research Unit, Plot 51-59 Nakiwogo Road, P. O Box 49, Entebbe, Uganda; 2 London School of Hygiene and Tropical Medicine, Keppel Street, London, WC1E 7HT UK

**Keywords:** Universal test and treat, Women at high-risk, Lost to follow-up, Loss to follow-up; sub-Saharan Africa

## Abstract

**Background:**

Immediate uptake of antiretroviral therapy (ART) after an HIV-positive diagnosis (Test and Treat) is now being implemented in Uganda. Data are limited on lost to follow-up (LTFU) in high-risk cohorts that have initiated ‘Test and Treat’. We describe LTFU in a cohort of women of high-risk sexual behaviour who initiated ART under “Test and Treat”.

**Methods:**

We performed a retrospective cohort study of participant records at the Good Health for Women Project (GHWP) clinic, a clinic in Kampala for women at high-risk of HIV-infection. We included HIV positive women ≥18 years who initiated ART at GHWP between August 2014 and March 2018. We defined LTFU as not taking an ART refill for ≥3 months from the last clinic appointment among those not registered as dead or transferred to another clinic. We used the Kaplan-Meier technique to estimate time to LTFU after ART initiation. Predictors of LTFU were assessed using a multivariable Cox proportional hazards model.

**Results:**

The mean (±SD) age of the 293 study participants was 30.3 (± 6.5) years, with 274 (94%) reporting paid sex while 38 (13%) had never tested for HIV before enrolment into GHWP. LTFU within the first year of ART initiation was 16% and the incidence of LTFU was estimated at 12.7 per 100 person-years (95%CI 9.90–16.3). In multivariable analysis, participants who reported sex work as their main job at ART initiation (Adjusted Hazards Ratio [aHR] =1.95, 95%CI 1.10–3.45), having baseline WHO clinical stage III or IV (aHR = 2.75, 95% CI 1.30–5.79) were more likely to be LTFU.

**Conclusion:**

LTFU in this cohort is high. Follow up strategies are required to support women on Test and Treat to remain on treatment, especially those who engage in sex work and those who initiate ART at a later stage of disease.

## Background

It is estimated that by the end of 2018, 23.3 million people were receiving antiretroviral therapy (ART) globally and that between 2000 and 2018, HIV-related deaths fell by 45% with 13.6 million lives saved due to ART in the same period [1]. In sub-Saharan Africa (SSA) access to ART has improved from 10.3 million people in 2015 to 12.9 million people by the end of 2018 [2]. In Uganda, by the end of 2018, it was estimated that 1.4 million people were living with HIV of whom 73% were on ART and an estimated 23,000 Ugandans had died of HIV-related illnesses [1]. In Uganda, the general population HIV prevalence has stabilised at 6% [3] while the prevalence among key populations (such as female sex workers [FSW]) is estimated to be over 30% [4]. Studies have shown that ART is not only improving quality of life and decreasing morbidity in those receiving treatment but that the public health impact of scaling up ART may contribute significantly to prevention efforts [5, 6]. Recent studies have shown that early ART initiation reduced transmission of HIV and that there is great potential of treatment for prevention [6–8]. Given that the risk of HIV infections is high for key populations [9], this scale-up in ART is particularly important to maximize the effectiveness of treatment as prevention in such populations [10]. The scale up among key populations may have an impact on the HIV epidemic, since it is estimated that in SSA they contribute between 7 and 30% of new infections [11, 12].

The scale-up of treatment can only be effective if people living with HIV remain on treatment following diagnosis and ART initiation, but retention in care remains a challenge in both high and low income settings [13]. With the current efforts aimed at initiating all HIV-positive individuals on immediate ART, regardless of CD4 and WHO clinical stage, in an approach called “Test and Treat” [14], retention in care is key if the full potential is to be realised including achieving the 90–90-90 targets especially the 3rd 90 which is the one mostly affected by retention [15].

Treatment has become more widely available, but few data have been published on rates of retention and predictors of loss to follow-up (LTFU) among people at high-risk of HIV-infection enrolled on immediate ART. Studies carried out in the general population have shown that LTFU ranged between 14 and 18% for the people living with HIV initiated on ART with some studies indicating that 41% of the LTFU occurred within the first 6 months of ART initiation [16–18]. We build on this work with a cohort of HIV positive women who started ART in a “Test and Treat” facility in Kampala, Uganda during the period of August 2014 to March 2018. We estimated the rate of LTFU and associated factors/predictors.

## Methods

### Study design

We performed a retrospective cohort study among women at high-risk of HIV infection, on ART, using records between August 2014 and March 2018.

### Study population and setting

This study was embedded within an existing cohort of women living with HIV, or at high risk of HIV infection, attending the Good Health for Women Project (GHWP) clinic in Kampala. The GHWP clinic was established in a peri-urban community in southern Kampala in 2008 under the then Medical Research Council/Uganda Virus Research Institute (MRC/UVRI), Uganda Research Unit on AIDS to study the epidemiology of HIV and sexually transmitted infections (STIs) and to implement HIV/STI prevention among high-risk women. Women attending the clinic engage in risky sexual behaviours such as sex with men for money, goods or favours; recruitment of women from commercial-sex hotspots has been described elsewhere [4]. All women were screened for eligibility and gave written consent before being enrolled in GHWP. Eligible women are then enrolled in the GHWP clinic irrespective of HIV status. The clinic offers routine HIV counselling and testing, syndromic management of STIs, family planning, antenatal care, free condoms, risk reduction counselling, counselling for excessive alcohol use, TB screening and treatment, ART and co-trimoxazole /dapsone preventive therapy. Enrolled women attend quarterly visits for HIV prevention and treatment services.

In August 2014, the GHWP clinic started the “test and treat” in accordance with new ART guidelines [19], which mandated that all people living with HIV should be initiated on ART irrespective of CD4 T-cell count and WHO clinical staging, as long as they were willing and ready to start treatment.

### Eligibility criteria

All HIV positive women who initiated immediate ART at the clinic were eligible for screening into this study. Participants were included in this study if they met the following eligibility criteria: HIV positive women ≥18 years and initiated ART between August 2014 and March 2018.

### Definition of study variables

#### High-risk

Women were classified as being at high-risk of HIV-infection if they were earning their living by exchanging sex for money, goods or other favors in lodges, on the street and around entertainment places. Women were also classified as being at high risk if they were illicit drug users, alcohol dependent and employed in entertainment facilities (for instance as waitress, massage attendants or entertainers/singers) where they may exchange sex for money after working hours, in order to supplement their income.

#### Study outcome

The study outcome was Lost to Follow-Up (LTFU) which we defined as not collecting ART refill for 3 months or longer from the last clinic appointment and not classified as dead or transferred for care to another clinic [20]. Women who had died or transferred their care to another clinic were censored at the time of their last visit and were not considered LTFU. The time to LTFU was calculated in months as the time interval between the date of ART initiation to the date of drop out (from ART initiation to first 3-month drop out), as recorded in the clinic ART database. All participants were followed until March 2018 unless they were LTFU, or had transferred or died before then.

#### Independent variables

Baseline characteristics at ART start were considered for this analysis. The sociodemographic characteristics considered were: participant’s age at ART initiation, marital status, highest level of education attained, current job, behavioural characteristics were; ever being involved in paid sex, alcohol consumption patterns were assessed by using the Alcohol Use Disorders Identification Test (AUDIT) questionnaire which is a standardised tool developed by WHO. It consists of 10 questions to assess alcohol use disorders in the past 12 months [21], and, Clinical characteristics included: baseline WHO clinical stage, baseline CD4 cell count (cells/μl), year of ART start and ever tested for HIV before enrolling at the clinic.

#### ART start and follow-up procedures

The GHWP clinic staff gave information sessions about the ‘Test and Treat’ guidelines to women and the benefits to their health and the wider community. Women were also taught and counselled on disclosure, drug reactions, the importance of treatment adherence, and then individually assessed for readiness to start ART using the Ministry of Health (MoH) ART readiness assessment form [19]. Using the MoH ART guidelines, the initial ART prescriptions were for 2 weeks, then 1 month followed by an evaluation to check for any ART side effects, and then three-monthly ART refills. Attending an ART refill visit entailed visiting the clinic, seeing a physician, and receiving counselling before getting drugs from the pharmacy. First-line ART regimens dispensed at the clinic combined two nucleoside reverse transcriptase inhibitors (Tenofovir or Zidovudine and Lamivudine or Emtricitabine) and one non-nucleoside reverse transcriptase inhibitor (Nevirapine or Efavirenz).

#### Follow up strategies for ART participants

The pharmacist generates the list of women expected to come for ART refill. On a weekly basis, the field team follows up the clients (makes reminder calls) i.e. phone calling and physical visits either at home or at their places of work (hotspots) reminding them of their pending appointments.

In situations where the client has no telephone contact or their phone is unavailable, the field worker contacts a peer educator to pass over the information to the respective client/s. In case, the client misses the appointment and there is no documented reason for missing (reasons like travelled to the village, or to the islands, abroad etc.), then the field worker retrieves the locator form for such client and try to look for her physically, sometimes with the help of the peer educator or an influential person at the hotspot e.g. the lodge manager. When found, the field worker encourages such client to come and finds out the reasons for missing the appointment. The process continues until the client is located and encouraged to attend or declared lost to follow up.

#### Sources of data

Data were collected as part of routine clinical care monitoring and for research purposes within the clinic. Data entered in the clinic database (MS Access, Microsoft, Seattle, CA, USA) captured sociodemographic, alcohol use and sexual behaviour, clinical and reproductive health characteristics. HIV monitoring data such as HIV test results and all ART data were entered in an OpenMRS version 1.6.3 database which was merged with the GHWP database. Data were collected during one-on-one private sessions held in clinic rooms with trained GHWP clinic counsellors. The pharmacy team captured data on ART refill dates, duration on ART, last ART refill dates and prescriptions. HIV antibody test results were recorded on laboratory source documents by laboratory staff while CD4 results were generated as printouts and entered manually into the OpenMRS database.

### Statistical analysis

The MS Access and OpenMRS databases were merged into one database, cleaned, and exported to STATA 15.0 (StataCorp, College Station, TX, USA) for analysis. The participants’ characteristics were summarised in descriptive terms such as mean, median, standard deviations (SD) or percentage, as appropriate. We used the Kaplan-Meier technique to estimate time to LTFU after ART initiation and present the estimated LTFU rates per 100 person-years for the study variables. LTFU rate was assessed within the first year of follow-up. Cox proportional hazards regression was used to determine independent predictors of LTFU among participants and these were expressed as estimated hazard ratios (HRs) with their corresponding 95% confidence intervals (CIs). In the bivariate proportional hazards analysis, variables which gave a Likelihood Ratio Test (LRT) *p*-value less than 0.25 were subsequently considered for the adjusted model. In adjusted modelling, variables whose p-value was less than 0.05 were considered independent predictors of LTFU. We tested the validity of the overall model assumption by running a global test of the proportional-hazards assumption after fitting the model. We considered age at ART initiation and prior HIV testing a priori confounders; these were included in the multivariable model regardless of their unadjusted *p* values.

We further compared those who started ART at GHWP and those who did not to see if there were significant differences between the two groups in terms of the selected socio-demographic and clinical characteristics.

### Sensitivity analyses

We conducted sensitivity analyses to address the issue of missing data on selected covariates. Participants who had missing data on some covariates were imputed using multivariate imputation by chained equations approach [22]. A sample of missing values were created, conditional on the distribution of the remaining covariates in the adjusted model. We assumed that the data were missing at random and carried out 10 rounds of multiple imputations; the final data for analysis after imputation were combined using Rubin’s rule [23]. We compared the results from the complete case analysis and those from the imputed model.

### Ethical considerations

The Uganda National Council for Science and Technology (HS 364) and Uganda Virus Research Institute-Research Ethics Committee approved the study. Written informed consent was obtained from all participants. Data were de-identified prior to analysis of the numerical identifiers that were used during the data collection.

## Results

**Fig. 1:**
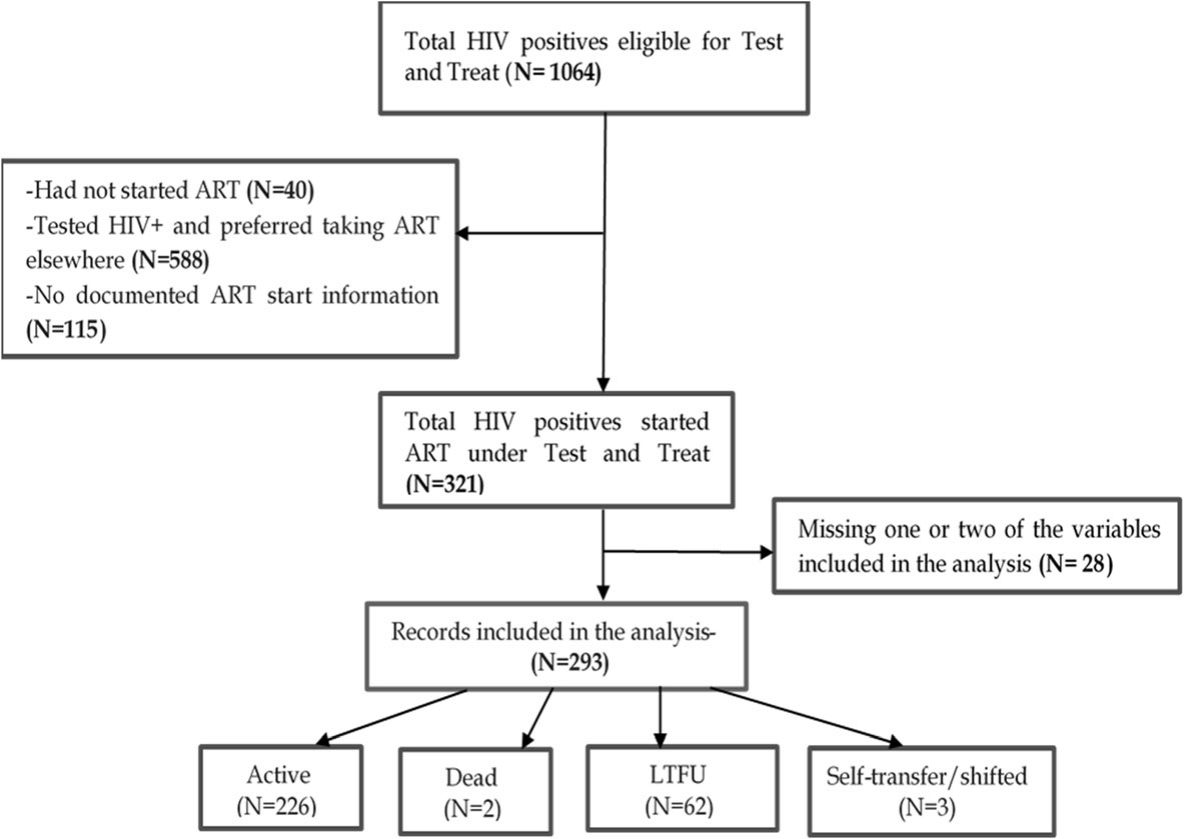
Screening Profile of Study Participants

Between August 2014 and March 2018, 3062 participants were cumulatively registered with GHWP of whom 1062 were HIV-positive and eligible for test and treat. During the study period, 44 more women who were originally negative became HIV positive and became eligible giving 1106 (44 + 1062) women. Of the 1106, 321 HIV-positive participants fully enrolled on “Test and Treat” of whom 293 (91.3%) who had complete records were included in the analysis. Details are shown in the screening profile (Fig. 1).

### Baseline characteristics of women in the “test and treat” programme

A total of 293 participants were included in the statistical analysis. The mean (±SD) age of study participants was 30.3 (± 6.5) years. Two hundred and fifty-five (87.0%) had been tested for HIV before enrolment in GHWP, 196(66.9%) were widowed or separated, 52(17.7%) were never married; 191(65.2%) were at WHO clinical stage I; 75(25.6%) were at WHO clinical stage II. One hundred ninety-seven (67.2%) participants reported sex work as their current job. Those who reported no sex work had other jobs including working in an entertainment facility, a hotel/guest house or food vending; 3.1% reported being unemployed. The median baseline CD4 (IQR) was 530 (348–757) cells/μl (Table 1).

**Table 1:** Baseline sociodemographic and clinical characteristics of the 293 study participants enrolled in the Test and Treat programme between August 2014 and March 2018 in Kampala Uganda

Characteristics	Category	Frequency ***N*** = 293 n (col%)	Did not start ART at GHWP ***N*** = 588 n (col%)	Chi-Square ***P***-value
Mean age (SD)		30.3 (6.5)	28.4 (6.1)	< 0.001
Age at enrolment (year)	< 25	50 (17.1)	178 (30)	< 0.001
	25–34	175 (59.7)	303 (52)	
	35+	68 (23.2)	107 (18)	
Marital Status	Widowed/Separated	196 (66.9)	425 (72)	< 0.001
	Married	45 (15.4)	36 (6)	
	Never married	52 (17.7)	127 (22)	
Current job	No sex work	96 (32.8)	136 (23)	0.002
	Sex worker	197 (67.2)	452 (77)	
Highest formal education level attained	No education	37 (12.6)	60 (10)	0.553
	Attended Primary school	177 (60.4)	363 (62)	
	Secondary school or higher	79 (27.0)	165 (28)	
Alcohol Consumption Patterns (AUDIT score)^a^	Low risk	113 (38.6)		
	High risk	119 (40.6)		
	Dependent	61 (20.8)		
Reported paid sex	Yes	274 (93.5)	575 (98)	0.001
	No	19 (6.5)	13 (2)	
Year of ART start^a^	2014	15 (5.1)		
	2015	75 (25.6)		
	2016	130 (44.4)		
	2017	73 (24.9)		
Baseline WHO stage^a^	WHO Stage I	191 (65.2)		
	WHO Stage II	75 (25.6)		
	WHO Stage III & IV	27 (9.2)		
Median CD4 count (IQR)		530 (348,757)		
Baseline CD4 count (cells/μl)^a^ Median (IQR)	0–350	74 (25.3)		
	Above 350	219 (74.7)		
Ever tested for HIV before enrolling at the clinic	Yes	255 (87)	560 (95)	< 0.001
	No	38 (13)	28 (5)	

*SD* Standard deviation, *IQR* Interquartile range; AUDIT Scores: 0–7 Low Risk, 8–19 High risk, ≥20 Dependent; ^a^No available data for the comparison group

Compared with those included in the analysis, those who did not initiate ART at GHWP were not significantly different in terms of highest level of education attained. Furthermore, those included in the analysis were older, a higher proportion were married, a lower proportion reported sex work as their main job and had fewer had a prior HIV test when compared to those who did not initiate ART at GHWP. These were significantly different (Table 1).

### Lost to follow-up and associated factors

**Fig. 2:**
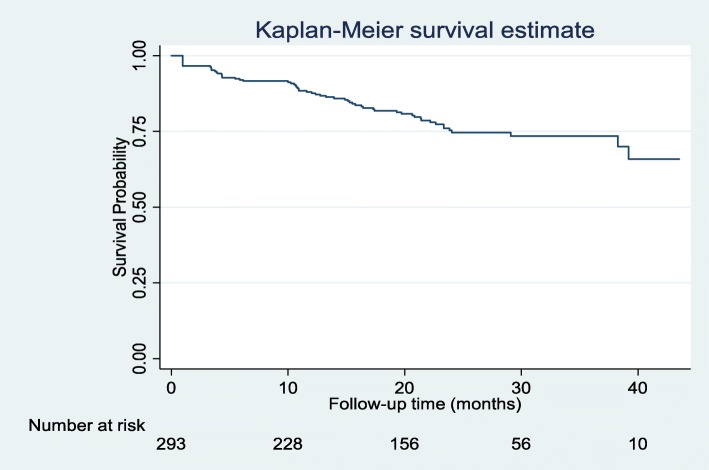
Kaplan-Meier plot showing LTFU of participants during the follow-up period

Of the 293 enrolled participants, 16% were LTFU within the first year of ART initiation. The median follow-up time was 1.77 years, with the longest follow-up being 3.6 years. At the end of the follow-up period, three records were censored due to known transfer out/shifted while two were due to death. We recorded a total of 488.15 person-years of follow up, and the estimated LTFU rate was 12.7 per 100 person-years (95% CI 9.90–16.3). Figure 2 illustrates the survival curve for the time to LTFU after ART initiation during the follow-up period. Close to 90% of the participants were still in care after 12 months of follow-up.

In the unadjusted analysis, LTFU was more likely among those who reported sex work as their main job at ART initiation compared to those who reported no sex work (uHR 1.94; 95% CI 1.03–3.43), and those who had baseline WHO clinical stage III or IV compared to those who had baseline WHO clinical stage I (uHR 2.97; 95% CI 1.50–5.86).

In the adjusted model, participants who reported sex work as their current job at ART start were at a higher risk of LTFU (aHR 1.95; 95%CI 1.10–3.45) while those who had baseline WHO clinical stage III or IV were at higher risk of LTFU (aHR 2.75; 95%CI 1.30–5.79) compared to those who had baseline WHO clinical stage I. (Table 2). The global test results showed *p* > 0.05 thus we do not have a violation of the proportional assumption.

**Table 2:**
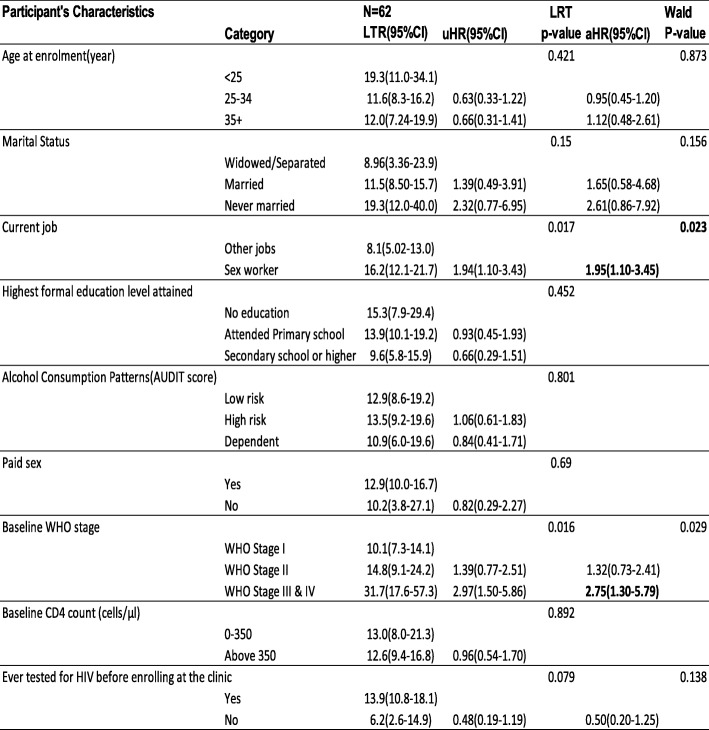
Multivariable analysis of predictors of Lost to follow-up among study participants enrolled in the Test and Treat programme between August 2014 and March 2018 in Kampala Uganda

^*^significant at *P* < 0.05; *HR* Hazard Ratio, *I* Confidence Interval, *LRT* Likelihood Ratio Test, *LTR* Estimated LTFU rate per 100 person-years, *uHR* Unadjusted Hazard Ratio, *aHR* Adjusted Hazard Ratio, *Wald* Wald Chi-square *P*-value; global test chi-square value = 6.90 and *p*-value = 0.548

### Sensitivity analysis results

A sensitivity analysis by imputing data of participants who had missing data on some covariates (*n* = 28) was done and the results were similar to when they were excluded.

## Discussion

In this study, we illustrate high rates of LTFU in a cohort of early adopter, test and treat recipients of Ugandan women at high risk of HIV-infection. Our study revealed that the overall LTFU rate was 12.7 per 100 person-years. The proportion of LTFU of 16% in the first year of ART initiation in this study is high compared to the overall results of the meta analysis of attrition among FSWs that was reported at 6% [24]. In studies carried out among high-risk populations in coastal Kenya, high rates of LTFU were found among men who have sex with men (MSM) (14.5/100 PYAR) which is consistent with our study and lower rates among women (10.4/100 PYAR) [25]. Other studies carried out in the general population in sub-Saharan Africa and elsewhere showed lower LTFU rates ranging between 7.1–11.6 [18, 26, 27]. Some of the variations in the incidence rates might be explained by the differences in the populations studied especially with those in the general populations, as our study was carried out among a high-risk population. For the studies carried out in a similar population, the differences might be explained by the follow-up strategies that are used to reach to these participants [28].

Women who reported sex work as their current job at the time of ART initiation were more likely to be LTFU compared to those who reported other jobs. Studies carried out in sub-Saharan Africa, the Caribbean and elsewhere have found that FSWs are highly mobile and because of this, it is hard for them to access secure housing, supportive networks and regular income, this makes it hard for them to start and stay on ART [24, 29, 30]. In the qualitative studies carried out among FSWs seeking ART/general care services, major concerns were rude remarks from providers, delay of services and potential for breach of confidentiality [30, 31]. Although we did not assess this, we assume that might explain why some FSWs are likely to get lost after initiating prompt ART. Many FSWs have a lifestyle which poses barriers to stabilise on treatment such as high alcohol intake, fear of being seen taking drugs which might result into losing their clients [32, 33].

There is a need to establish strategies targeting sex workers to improve access to ART services conveniently for example giving them special attention and according them respect and continued assurance of confidentiality when they come for drug refills and appointments. In addition to the close monitoring and intensive follow-up at the GHWP clinic, there is a need to have a sex worker-supportive environment through community engagements among FSWs so that universal access to HIV services and acceptable ART delivery strategies can be identified [34].

In our study, we found that women who had baseline clinical stage III or IV were more likely to get lost to follow-up compared to those with baseline clinical stage I. Our results are consistent with case-control studies that were carried out in Uganda, Ethiopia and in another study carried out in antiretroviral treatment (ART) clinics in Tanzania, Uganda and Zambia to assess predictors of attrition [35–37]. Though we did not trace participants in an advanced stage, studies done in SSA have shown that this group tends to die early [38] . However, our results contradict the findings of the study carried out in Ethiopia in a general population which found out that clinical WHO stage III was not associated with LTFU [39]. This difference might be due to the difference in study settings where this study in Ethiopia was carried out in governmental health institutions (3 health centres and 2 Hospitals) which provide ART service for the town and surrounding population while our study was carried out in a dedicated clinic that follows high risk women. Therefore, this group needs to be targeted and encouraged to always stay in care in order to achieve the 90–90-90 targets.

### Study limitations

The main limitation is that we had 62 LTFU events from our study, we believe a bigger study might have detected further risk factors. We believe the fewer events might affect our generalizability.

Another limitation was missing data on some of the variables that were included in the analysis. Of the 321 that enrolled on “test and treat” at GHWP clinic, only (91.3%; 293/321) had all the data available for analysis on the selected variables. We however did the sensitivity analysis which showed no differences in the results with all the missing data imputed.

We used secondary data; some of the data that may influence the risk of LTFU such as social support were not available. We believe such explanations would inform future strategies of targeting groups of high-risk women on ART. Notwithstanding these constraints, we believe that our results will give an imperative understanding of the magnitude and the factors associated with LTFU among high-risk cohorts in the test and treat era.

### Recommendation for further research

We recommend a qualitative research study among the high-risk cohorts by exploring the barriers and challenges of this group in regard to LTFU. This should focus on other behavioural characteristics.

## Conclusion

Our findings report a high rate of lost to follow-up in this cohort. Follow up strategies are required to support women who initiate Test and Treat to, especially those who engage in sex work as their main job and those who initiate ART at a later stage of disease.

## Data Availability

Data used for this paper will not be shared publically but if there is a need for this data, then data can be shared by following the data sharing policy of MRC/UVRI & LSHTM Uganda research unit. This policy can be access through this link https://www.mrcuganda.org/publications/data-sharing-policy.

## Abbreviations

aHR: Adjusted Hazard ratio
ART: Anti-retroviral treatment
AUDIT: Alcohol Use Disorders Identification Test
CD4: Cluster of differentiation 4
CI: Confidence Interval
FSWs: Female sex workers
GHWP: Good Health for Women Project
HIV: Human Immunodeficiency Virus
LRT: Likelihood Ratio Test
LTFU: Lost to Follow-up
MoH: Ministry of Health
MRC/UVRI and LSHTM: Medical Research Council/ Uganda Virus Research Institute and London School of Hygiene and Tropical Medicine
MSM: Men who have sex with men
PYAR: Person years at risk
SD: Standard deviation
SSA: Sub-Saharan Africa
STIs: Sexually Transmitted Infections
uHR: Unadjusted Hazard ratio
UNCST: Uganda National Council for Science and Technology
UVRI-REC: Uganda Virus Research Institute-Research Ethics Committee
WHO: World Health Organization
